# Capturing and releasing of hepatocellular carcinoma EpCAM+ and EpCAM- circulating tumor cells based on photosensitive intelligent nanoreactor

**DOI:** 10.3389/fbioe.2024.1443843

**Published:** 2024-08-30

**Authors:** Zhifang Mao, Meng Hu, Qinglin Shen

**Affiliations:** ^1^ Department of Oncology, Jiangxi Provincial People’s Hospital, the First Affiliated Hospital of Nanchang Medical College, Nanchang, China; ^2^ Institute of Clinical Medicine, Jiangxi Provincial People’s Hospital, the First Affiliated Hospital of Nanchang Medical College, Nanchang, China; ^3^ Jiangxi Province Key Laboratory of Immunity and Inflammation, Nanchang, China

**Keywords:** photosensitive intelligent nanoreactor, circulating tumor cells, EpCAM+, EpCAM-, hepatocellular carcinoma

## Abstract

Epithelial cell adhesion molecule negative circulating tumor cells (EpCAM- CTCs) and EpCAM positive CTCs (EpCAM + CTCs) have different biological characteristics. Therefore, the isolation of EpCAM + CTCs and EpCAM- CTCs is a new strategy to study the heterogeneity of tumor cells. The azobenzene group (Azo) and cyclodextrin (CD) composite system forms a photosensitive molecular switch based on the effect of external light stimulation. We used the technology of specifically capturing CTCs using anti-EpCAM and aptamers functionalized nanochips. Both anti-EpCAM and aptamers can be connected to Azo through the 1-ethyl-3-(3-dimethylaminopropyl) carbodiimide/N-hydroxysuccinimide (EDC/NHS) modification process. Therefore, we assume that a photosensitive intelligent nanoreactor (PSINR) modified with anti-EpCAM can be used to capture EpCAM + CTCs; Utilizing the characteristics of aptamer and ligand binding, a PSINR modified with aptamer is used to capture EpCAM- CTCs; Then, two PSINRs were separated and stimulated with light to release EpCAM + CTCs and EpCAM- CTCs, respectively. Based on the isolation the EpCAM + CTCs and EpCAM- CTCs, we expected to reveal the key biological mechanisms of tumor recurrence, metastasis and drug resistance, and make the individualized treatment of liver cancer more targeted, safe and effective, and provide a new basis for the final realization of accurate and individualized treatment of tumors.

## 1 Introduction

CTC (Circulating Tumor Cell) is a general term for various types of tumor cells present in the peripheral blood (Deng et al., 2022). CTC detection detects the trend of changes in the type and number of CTCs by capturing and detecting the presence of CTCs in peripheral blood, so as to monitor tumor dynamics in real time, evaluate treatment effects, and achieve real-time individual treatment (Wang et al., 2023). Malignant tumors are spread through the bloodstream to other organs of the body, and tumor metastasis is the main cause of death in cancer patients. Tumor cells invade the surrounding tissues of the primary tumor cells, enter the blood and lymphatic vessels, form CTCs, and transport them to distal tissues, where they exude and adapt to the new microenvironment, and finally “seed”, “proliferate”, “colonize” and form metastases (Follain et al., 2020). Therefore, the early detection of CTCs in blood has an important guiding role in the prognosis judgment, efficacy evaluation and individualized treatment of patients (Ma et al., 2023).

Epithelial cell adhesion molecule (EpCAM) negative CTCs (Li et al., 2024) (EpCAM-CTCs) refer to CTCs that do not express EpCAM, and EpCAM- CTCs and EpCAM positive CTCs (EpCAM + CTCs) have different biological characteristics, that is to say, tumor heterogeneity. EpCAM + CTCs patients had a worse overall and progression-free survival (Franken et al., 2023), and EpCAM- CTCs had the low metastatic potential (Lampignano et al., 2017b). Therefore, the isolation of EpCAM + CTCs and EpCAM- CTCs is a new strategy to study the heterogeneity of tumor cells.

The principle of capture and release of CTCs includes the capture method based on biophysical principles (Bartosik et al., 2020), which refers to the use of filtration, centrifugation, electrophoresis, inertial focusing, acoustic wave, etc., and matrix materials based on the principle of biological affinity mainly include magnetic beads, microfluidic chips and materials with micro and nano structures. One of the main principles of capturing CTCs is the specific binding of Epithelial cell adhesion molecule (EpCAM) antigen expressed on the surface of CTCs to the antibody, but this method mainly captures and releases EpCAM + CTCs, resulting in the neglect of EpCAM- CTCs. Therefore, there is an urgent need to find a method to capture and release both EpCAM+ and EpCAM- CTCs simultaneously.

**FIGURE 1:**
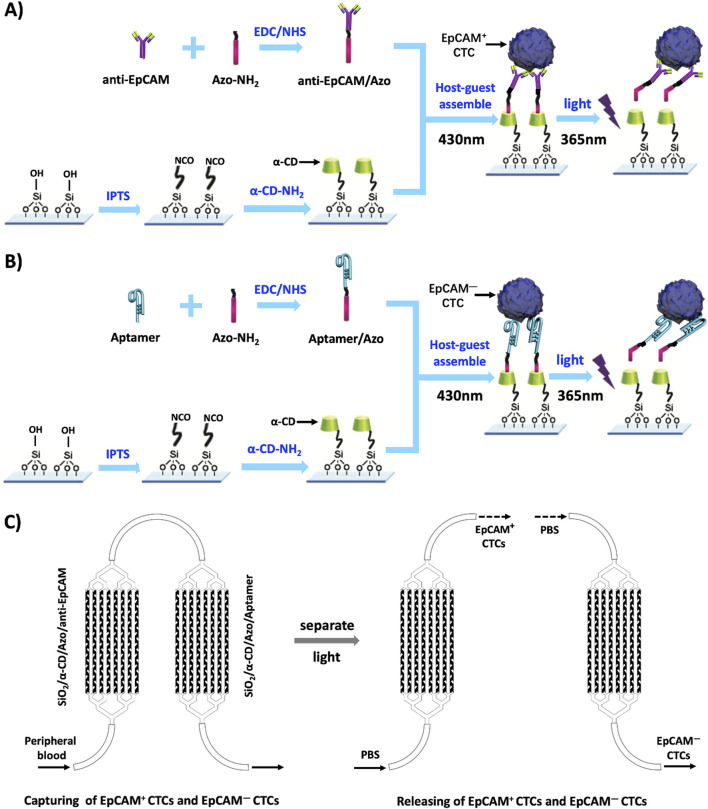
A schematic diagram of the capture and release of EpCAM + CTCs and EpCAM- CTCs. Notes: **(A)** A schematic diagram of the capture and release of EpCAM + CTCs by a photosensitive smart nanoreactor modified with anti-EpCAM. **(B)** A schematic diagram of the capture and release of EpCAM- CTCs by a photosensitive smart nanoreactor modified with Aptamer. **(C)** The flow chart of the capture and release of EpCAM + CTCs and EpCAM- CTCs by the PSINR. Abbreviations: α-CD-NH2:Amino - α- Cyclodextrin; Azo-NH2: p-aminoazobenzene; IPTS:1-Isocyanate propyltriethoxysilane; EDC/NHS: (1- (3-dimethylaminopropyl) -3-ethylcarbodiimide hydrochloride)/N- hydroxysuccinimide.

In recent years, the potential applications of intelligent nanomaterials in controllable catalysis, drug delivery, sensor systems, and intelligent nanodevices have attracted researchers’ interest (Ding et al., 2023; Zheng et al., 2023; Bag et al., 2024; Xu et al., 2024; Zhang et al., 2022b; Liao et al., 2022). More importantly, the application of these nanomaterials or nanotechnology has brought tremendous progress to the diagnosis and treatment of tumors (Liao et al., 2020; Liang et al., 2024; Zhang et al., 2022a). According to the stimulation methods, intelligent nanomaterials can be classified into temperature sensitive, pH sensitive, photosensitive, electromagnetic sensitive, and pressure sensitive types (Dong et al., 2019). The characteristics of intelligent nanomaterials provide us with an opportunity to solve this problem. Through literature study, we learned that the azobenzene group (Azo) can undergo cis trans isomerization under the effect of external light stimulation, thus reversible recombination (430 nm) and decomposition (365 nm) processes with cyclodextrin (CD) by host–guest interactions (Liu et al., 2017), namely, the Azo group and α- CD composite system forms a photosensitive molecular switch, which is then branched onto a Silicon dioxide (SiO_2_) chip to form a photosensitive molecular switch. In the early stage, we studied the technology of specifically capturing CTCs using EpCAM antibodies and aptamers functionalized nanochips (Shen et al., 2013; Shen et al., 2016; Hou et al., 2013). Both anti-EpCAM and aptamers can be connected to Azo-NH2 through the 1-ethyl-3-(3-dimethylaminopropyl) carbodiimide/N-hydroxysuccinimide (EDC/NHS) modification process (Yavari et al., 2023; Song et al., 2019). Therefore, we assume that a PSINR modified with anti-EpCAM can be used to capture EpCAM + CTCs; Utilizing the characteristics of aptamer and ligand binding, a PSINR modified with aptamer is used to capture EpCAM- CTCs; Then, two PSINRs were separated and stimulated with light to release EpCAM + and EpCAM- CTCs, respectively, as shown in Figure 1.

## 2 Methods

### 2.1 Screening of aptamers with high specificity and high affinity for EpCAM-hepatocellular carcinoma (HCC) tumor cells

#### 2.1.1 DNA library selection

First, a 76-base DNA library with nucleotide sequences is chemically synthesized, which contains a central sequential 36-base random sequence and a conventional PCR primer region of 20 nucleotides on either side (i.e., 5′-TAC CTC TGA CAC ACG AG (36 nt) CTC ATG GAC GTG CAG CTG AG-3′). The synthesis of libraries and primers and purification by high performance liquid chromatography were completed by Dalian Takara Bioengineering Co., Ltd.

#### 2.1.2 Cell SELEX for aptamer screening

##### 2.1.2.1 EpCAM- SMMC-7721 cell sorting

SMMC-7721 and Jurkat cells were obtained from American Type Culture Collection (Manassas, VA, United States). According to the cell density, 1 × 10^3^ SMMC-7721 cells were added to 1 mL of culture medium after dilution, 10 μL of EpCAM-Fluorescein Isothiocyanate (FITC) antibody was added, the antibody and incubated cells were fully resuspended and mixed, stored in a 4°C freezer protected from light, and stained for 30 min, invert and mix every 5 min to prevent cells from sinking and uneven staining. The stained SMMC-7721 was then sorted by FACScan to II flow cytometry to screen out EpCAM + SMMC-7721 cells.

Collect the remaining liquid, centrifuge at 1,000 rpm for 5 min, discard the supernatant, resuspend the washed cells in pre-chilled PBS at 4°C once to remove excess antibodies to EpCAM-FITC that have not bound to the cells, add 1 mL of PBS again to resuspend the washed cells once, centrifuge at 1,000 rpm for 5 min, discard the supernatant, and obtain EpCAM- SMMC-7721 cells as subsequent target cells.

##### 2.1.2.2 Cell-SELEX technology screened aptamers with high affinity for EpCAM- SMMC-7721 cells

EpCAM- SMMC-7721 was a positive sieve cell, and human normal liver epithelial cell QSG-7701 was a reverse sieve cell.

First, the 6-nM DNA library was added to 500 μL of binding buffer (0.1 mg/mL of yeast tRNA, 1 mg/mL of BSA, 4.5 g/L glucose, and 5 mM of MgCl_2_ in PBS) and thawed at 95°C for 5 min, cooled on ice for 10 min, and then kept at room temperature for 5 min to obtain a pool of single-stranded DNA. The single-stranded DNA pool was then incubated with the EpCAM- SMMC-7721 cells screened in step (1) in a cell culture dish (5 cm diameter, Corning, United States) in 20% FBS for 90 min at 37°C。 After incubation, cells were washed twice with wash buffer (4.5 g/L of glucose and 5 mM of MgCl_2_ dissolved in PBS), scraped off with a cell scraper for collection, then transferred to a 1.5 mL tube for centrifugation washes 3 times, then heated at 95°C for 10 min to separate the DNA sequences bound to the surface of the target cells (EpCAM- SMMC-7721 cells), the supernatant was collected, and finally the supernatant was collected for the first PCR amplification, using primer sequences (forward primer:5′-FITC-TAC CTC TGA CAC ACG AG -3'; Reverse primers:5′-Biotin-CTC AGC TGC ACG TCC ATG AG-3′)to obtain the first amplification PCR product, and then 5 μL PCR of the first amplification product was taken as a template, and the second PCR was performed using the primers and reaction conditions of the first PCR reaction, and the double-stranded PCR product was separated on a 4% agarose gel.

The two PCR reaction procedures were: hot start at 95°C for 3 min and 1 cycle, denaturation at 94°C for 30 s, annealing at 59°C for 30 s, extension at 72°C for 20 s, 10–18 cycles.

The double-stranded PCR product was cleaved into single strands with agarose beads modified with streptavidin (Amersham Biosciences, United States), washed with PBS, and then denatured with 0.2 M of NaOH. The lysed single-stranded DNA solution is then passed through a Nap-5 desalting column (GE Healthcare, United States). Pools of single-stranded DNA labeled with selected FITCs were collected for the next round of screening and flow cytometry analysis.

In order to obtain aptamers with high affinity and specificity, the selective pressure is progressively increased by progressively shortening the incubation time with target cells (from 1.5 to 0.5 h) and by increasing the incubation time with control cells (from 1 to 1.5 h). After multiple rounds of screening, a concentrated single-stranded DNA library was obtained, which was sequenced with unmodified primers before amplification and cloned into *E. coli* (Takara, China) with TA cloning kit.

The sequencing results showed that 5 ligands were selected and named SHT1, SHT2, SHT3, SHT4, and SHT5, respectively. The nucleotide sequences of SHT1, SHT2, SHT3, SHT4, and SHT5 are shown in sequence from SEQ ID NO.1 to SEQ ID NO.5(The details as shown in the results).

The structure of these 5 aptamers was analyzed by DNAMAN software, and 10 truncated aptamers were obtained by truncating part of the sequences (nucleotide sequences are shown in SEQ ID NO.6-SEQ ID NO.15, the details as shown in the results). The truncated aptamers have different secondary structures, but most have a stem-loop structure. Flow cytometry measured the binding capacity of each aptamer and calculated the equilibrium dissociation constant (Kd value) of each aptamer. Aptamers (abbreviated as Apt-HCC-Neg) that can bind specifically to EpCAM- SMMC-7721 cells were screened.

##### 2.1.2.3 Flow cytometry to detect the equilibrium dissociation constant (Kd value)

To detect the enriched pool of single-stranded DNA and to assess the binding affinity (Kd value characterization) of the screened aptamer, single-stranded DNA labeled by FITC is detected by flow cytometry after different rounds. Specifically, it includes the following steps:

Aptamers at gradient concentrations (e.g., 0 nM, 10 nM, 25 nM, 100 nM, 200 nM, etc.) were incubated in 200 μL of binding buffer with 20% FBS and 3 × 10^5^ positive sieve cells for 60 min at 37°C to obtain aptamer solutions for flow cytometry.

Flow cytometry uses a distracting DNA sequence as a control. Both control and EpCAM- SMMC-7721 sequences were used. The average fluorescence intensity of 10^4^ cells with different aptamer and control DNA sequences was obtained. Detection of the pool of DNA enriched during SELEX, the final concentration of FITC-labeled single-stranded DNA is 500 nM. Further evaluation of binding affinity, the final concentrations of FITC-labeled aptamer solutions were 0 nM, 10 nM, 25 nM, 100 nM, 200 nM. Adherent cells are detached from the Petri dish with 0.02% EDTA.

##### 2.1.2.4 Verification of high specificity of the screened Apt-HCC neg

To verify that the screened aptamer binds specifically to EpCAM- SMMC-7721 and is bound to the cell membrane, we designed an experimental group and two control groups.

Experimental group: EpCAM- SMMC-7721 cells bind to FITC-labeled streptavidin and Apt-HCC-Neg;
Control group 1: EpCAM- SMMC-7721 cells with FITC-labeled streptavidin;
Control group 2: Jurkat cells were conjugated with FITC-labeled streptavidin and Apt-HCC-Neg, and the nuclei of each group were stained with DAPI, and then the fluorescence staining of each group was observed under a fluorescence microscope.

### 2.2 Preparation method of PSINR

#### 2.2.1 Isocyanate esterification on the surface of SiO_2_

The SiO_2_ chip was soaked in an appropriate amount of anhydrous tetrahydrofuran (THF), an appropriate amount of isocyanate propyltriethoxysilane (IPTS) was added, and the reaction was carried out at 40 °C under the protection of N_2_ for 8 h.

#### 2.2.2 SiO2 chip surface modification α-CD

The SiO_2_ chip modified by α-CD were obtained by soaking the SiO_2_ chip on the surface isocyanate in α-CD-NH_2_ solution for 24 h at room temperature, and then taking out the SiO_2_ chip after the reaction, washing them repeatedly with deionized water and drying them with N_2_.

#### 2.2.3 Preparation of azobenzene modified anti EpCAM and Apt-HCC neg

Take 200 μL anti EpCAM or Apt HCC Neg dissolved in 3 mL of deionized water, add 500 μ L’s 1- (3-dimethylaminopropyl) -3-ethylcarbodiimide hydrochloride (EDC) and N-hydroxysuccinimide (NHS) (in equal amounts and volumes) were stirred at 4 °C for 24 h. Afterwards, an appropriate amount of 3 nM para aminoazobenzene was added, and the reaction was further stirred at 4 °C for 24 h. Then, the temperature was raised to room temperature and the reaction continued for 24 h to obtain anti EpCAM/Azo or Apt HCC Neg/Azo modified with azobenzene.

#### 2.2.4 Host-guest interaction to construct a PSINR

Under dark conditions α- CD modified SiO_2_ chips can be soaked in azobenzene modified antibody or aptamer solution (anti EpCAM/Azo or Apt HCC Neg/Azo) for 24 h to construct a PSINR SiO_2_ through host guest interaction- α- CD/Azo anti EpCAM or SiO_2_- α- CD/Azo Apt HCC Neg.

### 2.3 Light stimulated PSINR captures and releases EpCAM + CTCs and EpCAM CTCs

#### 2.3.1 Specific capture and photosensitive release of EpCAM + CTCs and EpCAM- CTCs

Dilute 100 SMMC-7721 cells according to cell density and add them to 500 μL. After passing through the PSINRs modified with anti EpCAM and Aptamer at speeds of 0.5 mL/h, 1 mL/h, 2 mL/h, 4 mL/h, and 8 mL/h in culture medium, respectively, EpCAM + CTCs and EpCAM- CTCs were obtained, respectively, and then the two photosensitive smart nanoreactors were separated, and then the two photosensitive smart nanoreactors were irradiated with ultraviolet light, and then 500 μL was used Rinse the two chips 3 times in PBS, collect the flushed liquid, centrifuge, remove the supernatant, and the following ones are EpCAM + CTCs and EpCAM- CTCs, respectively.

#### 2.3.2 Identification of EpCAM + CTCs and EpCAM- CTCs

For the SiO_2_-α-CD/Azo-anti-EpCAM PSINR, we used the three-color principle of FITC-labeled EpCAM antibody and Cyanine5 (Cy5)-labeled cluster of differentiation 45 (CD45) antibody and 4′,6-diamidino-2-phenylindole (DAPI) to distinguish the non-specifically captured white blood cellsWBCs), and for the SiO_2_-α-CD/Azo-Apt-HCC-Neg PSINR. The laser power of fluorescence microscopy is 1 mW, and the exposure time is 20 s. We used the three-color principle based on Vimentin-conjugated Apt-HCC-Neg and Cy5-conjugated anti-CD45 anti and DAPI to distinguish non-specifically captured WBCs. The criteria for determining EpCAM + CTCs, EpCAM- CTCs, and WBC are as follows:EpCAM + CTCs:DAPI+/FITC+/Cy5-,WBC:DAPI+/FITC-/Cy5+; EpCAM- CTCs: DAPI+/Vimentin+/Cy5-, WBC: DAPI+/Vimentin-/Cy5+。

## 3 Results and disscusion

### 3.1 Cell-SELEX screened aptamers and equilibrium dissociation constants (Kd) for aptamers

To achieve better CTCs’ capture efficiency, we used cell SELEX technology to screen high affinity aptamers. We selected and named five Sequences from SEQ ID NO.1 to SEQ ID NO.5, and 10 truncated aptamers were obtained by truncating part of the sequences (nucleotide sequences are shown in SEQ ID NO.6-SEQ ID NO.15. The details can be seen in Table 1.

**TABLE 1:** The sequences of Cell-SELEX screened aptamers.

Sequence NO.	Sequence
SEQ ID NO. 1	5′- TAC​CTC​TGA​TGA​CAC​ACG​AGG​CGC​CAC​GGC​CGC​ATC​ATG​TGA​CTC​ATC​TAC​GCG​AAG​GTA​GCC​GTA​ATC​CCT​CAT​GGA​CGT​GCA​GCT​GAG-3′
SEQ ID NO. 2	5′- TAC​CTC​TGA​TGA​CAC​ACG​AGC​ATC​CAC​GGT​GCC​ATC​ATG​TGA​CTC​ATC​TAC​GCG​AAG​GTA​GTA​GTA​ACA​GCT​CAT​GGA​CGT​GCA​GCT​GAG-3′
SEQ ID NO. 3	5′- TAC​CTC​TGA​TGA​CAC​ACG​AGC​ATC​CAC​GGC​CGC​ATC​ATG​TGA​CTG​CGC​TAC​GCG​AAG​GTA​GAC​GTA​AAG​CCT​CAT​GGA​CGT​GCA​GCT​GAG-3′
SEQ ID NO.4	5′- TAC​CTC​TGA​TGA​CAC​ACG​AGC​ATC​CAA​TCC​CGC​ATC​ATG​TGA​CTC​ATC​TAC​GAC​AAG​GTA​GCC​GAG​CTC​CCT​CAT​GGA​CGT​GCA​GCT​GAG-3′
SEQ ID NO. 5	5′- TAC​CTC​TGA​TGA​CAC​ACG​AGC​ATA​ATC​GGC​CGC​ATC​ATG​TGA​CTC​ATC​TAC​GAC​AAG​GTA​GCC​GAC​GTC​CCT​CAT​GGA​CGT​GCA​GCT​GAG-3′
SEQ ID NO. 6	5′- TAC​CTC​TGA​TGA​CAC​ACG​AGA​TCC​CAC​GGA​ATC​ATC​ATG​TGA​ATC​ATC​TAC​GAC​AAC​TCA​TGG​ACG​TGC​AGC​TGA​G-3′
SEQ ID NO. 7	5′- TAC​CTC​TGA​TGA​CAC​ACG​AGG​CAC​CAG​CAC​CGC​ATC​ATA​CGA​CTC​ATC​TAC​GAG​GCC​TCA​TGG​ACG​TGC​AGC​TGA​G-3′
SEQ ID NO. 8	5′-TAC​CTC​TGA​TGA​CAC​ACG​AGC​ATA​GCC​GGC​CGC​ATG​CGG​TGA​CTC​ATC​TAC​GAT​GCC​TCA​TGG​ACG​TGC​AGC​TGA​G-3′
SEQ ID NO. 9	5′-TAC​CTC​TGA​TGA​CAC​ACG​AGT​GCC​CAC​GGA​TAC​ATC​ATC​GCA​CTC​ATC​TAA​CGC​AAC​TCA​TGG​ACG​TGC​AGC​TGA​G-3′
SEQ ID NO. 10	5′- TAC​CTC​TGA​TGA​CAC​ACG​AGG​CGC​CAC​GGC​CGA​TCC​ATG​TGG​CAC​ATC​TAC​GAC​AGC​TCA​TGG​ACG​TGC​AGC​TGA​G-3′
SEQ ID NO. 11	5′- TAC​CTC​TGA​TGA​CAC​ACG​AGC​ATC​GCC​GGA​CGC​ATC​ATG​TGA​CTG​CGC​TAC​GAT​AGC​TCA​TGG​ACG​TGC​AGC​TGA​G-3′
SEQ ID NO. 12	5′- TAC​CTC​TGA​TGA​CAC​ACG​AGA​CGC​CAG​CGC​CGC​ATT​AGG​TGT​AGC​ATC​TAC​GAG​CGC​TCA​TGG​ACG​TGC​AGC​TGA​G-3′
SEQ ID NO. 13	5′- TAC​CTC​TGA​TGA​CAC​ACG​AGC​CGT​AGC​GGA​CGC​ATC​ATG​TGT​GCC​ATG​GCC​GAC​AAC​TCA​TGG​ACG​TGC​AGC​TGA​G-3′
SEQ ID NO. 14	5′- TAC​CTC​TGA​TGA​CAC​ACG​AGC​GCC​CAC​ATA​CGC​ATC​ATC​CGA​CTC​ATC​TAC​GAG​GCC​TCA​TGG​ACG​TGC​AGC​TGA​G-3′
SEQ ID NO. 15	5′-TAC​CTC​TGA​TGA​CAC​ACG​AGC​ATG​CGC​GGA​TCC​ATG​CGG​TGA​CTC​ATC​TAC​GCC​AGC​TCA​TGG​ACG​TGC​AGC​TGA​G-3′

As can be seen from Table 2, the aptamers can specifically bind to EpCAM- SMMC-7721 cells, and have strong binding and high specificity, among which the equilibrium dissociation constant (Kd) of SEQ ID NO.9 is the lowest and the affinity is the largest.

**TABLE 2:** Detection of equilibrium dissociation constant of the selected aptamers.

Sequence NO.	Equilibrium dissociation constant (Kd) (nM)
SEQ ID NO. 6	237.6
SEQ ID NO. 7	265.2
SEQ ID NO. 8	283.4
SEQ ID NO. 9	198.3
SEQ ID NO. 10	367.8
SEQ ID NO. 11	326.4
SEQ ID NO. 12	275.3
SEQ ID NO. 13	342.1
SEQ ID NO. 14	238.9
SEQ ID NO. 15	356.2

### 3.2 Verification of high specificity of the screened Apt-HCC neg

**FIGURE 2:**
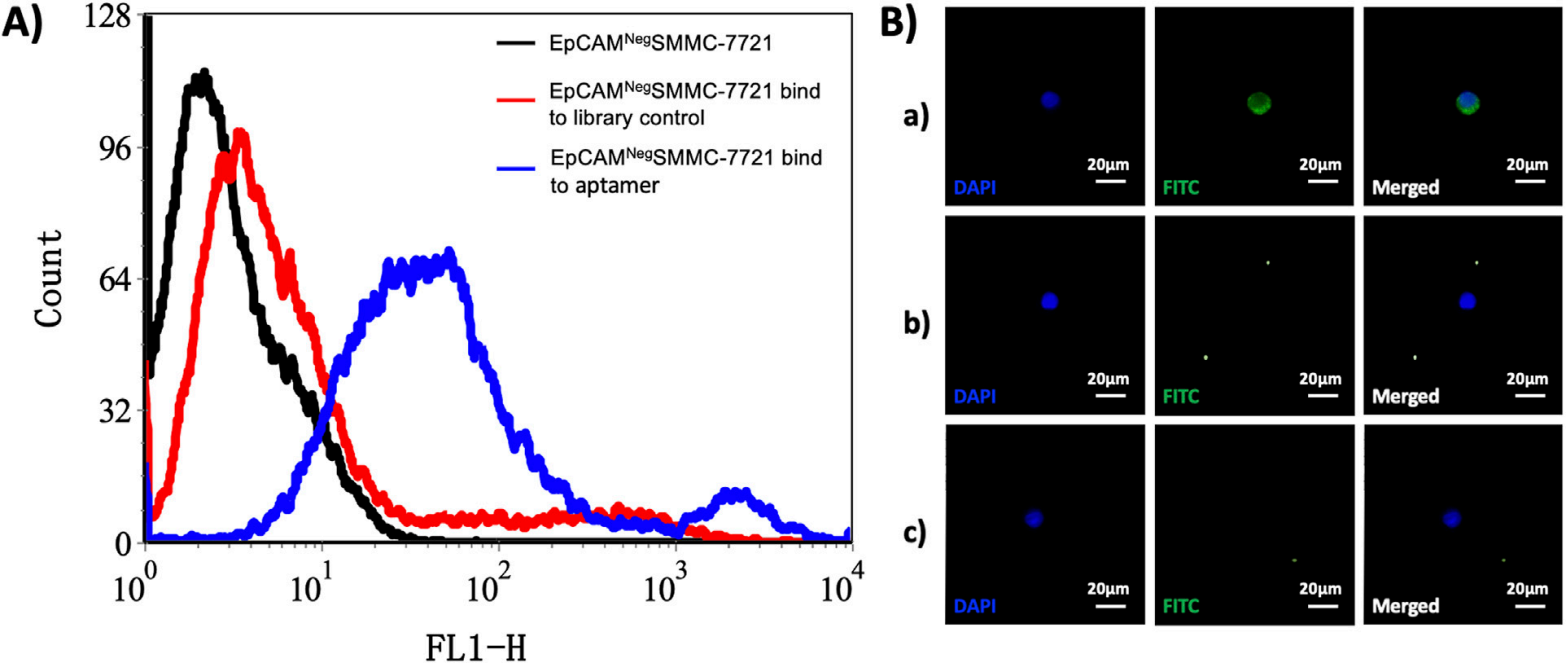
Verification of high specificity of the screened Apt-HCC Neg. Notes: **(A)** Flow cytometry detection of EpCAM- SMMC-7721 bound to FITC-labeled single-stranded DNA. **(A)** Black line: EpCAM- SMMC-7721 unbound single-stranded DNA; red line: EpCAM- SMMC-7721 cells bind to library control single-stranded DNA, blue line: EpCAM- SMMC-7721 cells bound to FITC-labeled single-stranded DNA; **(B)** To verify the specific binding of Apt-HCC-Neg to EpCAM- SMMC-7721 cells a) In the experimental group, EpCAM- SMMC-7721 cells bind to FITC-labeled streptavidin and Apt-HCC-Neg; b) Control group 1: EpCAM- SMMC-7721 cells were conjugated with FITC-labeled streptavidin; c) Control group 2: Jurkat cells were conjugated to FITC-labeled streptavidin and Apt-HCC-Neg (x200).

We applied the flow cytometry and the fluorescence staining to test and verify the screened Apt-HCC Neg with high specificity (The details can be seen in Figure 2A). To verify the high specificity of the screened Apt-HCC Neg, and is bound to the cell membrane,we used the Jurkat cells as a control (The details can be seen in Figure 2B). The superior performance of Apt-HCC Neg proves the feasibility of our screening process. The screened Apt-HCC Neg is the most critical substance for capturing EpCAM negative CTCs, and further confirming the reliability of our hypothesis.

### 3.3 Identification of EpCAM + CTCs and EpCAM- CTCs by immunofluorescence staining

**FIGURE 3:**
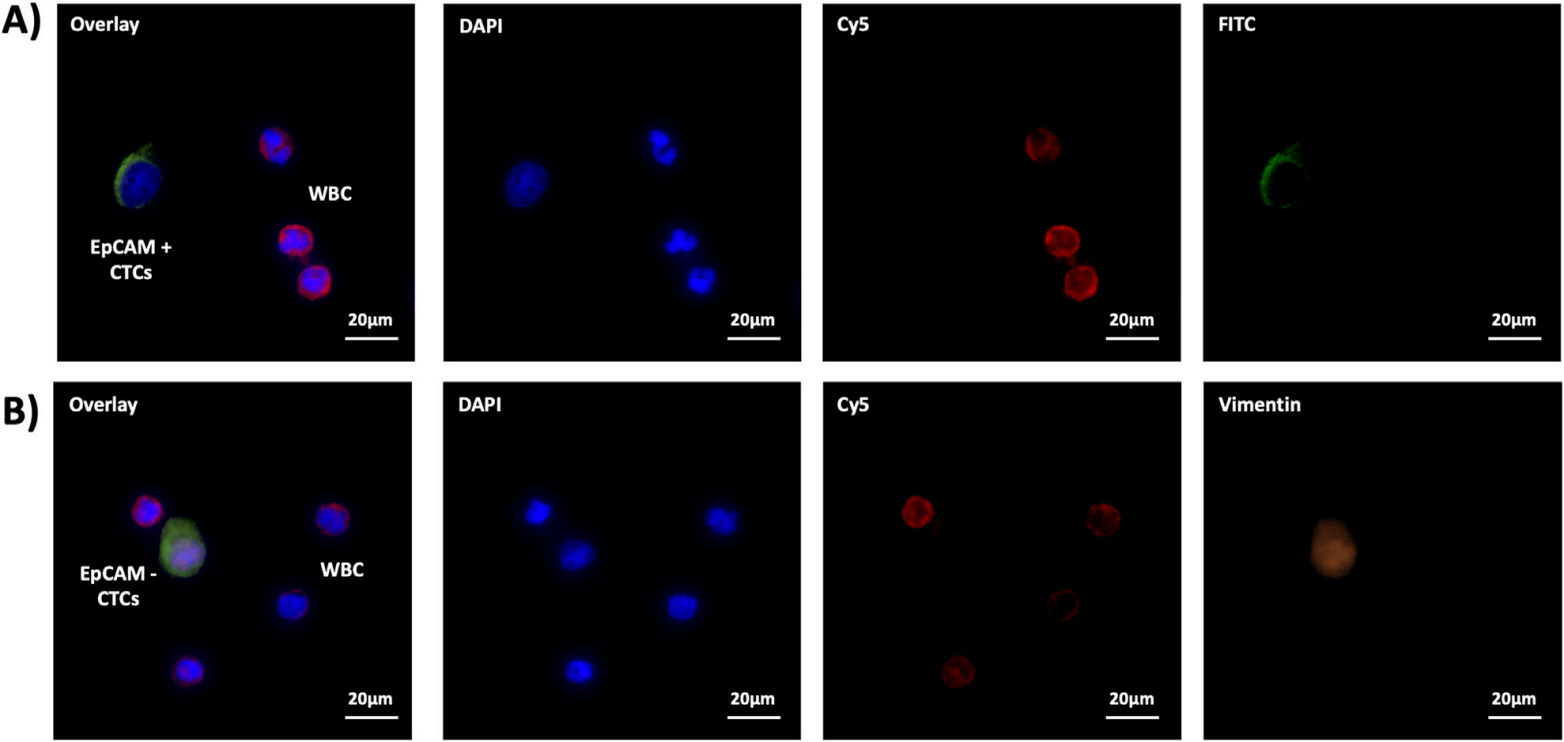
The immunofluorescence staining of **(A)** EpCAM+ CTCs and **(B)** EpCAM- CTCs.

To verify the performance of PSINR, we used the three-color principal immunofluorescence staining to identify EpCAM + CTCs and EpCAM- CTCs, and distinguish the non-specifically captured WBCs.The immunofluorescence staining results indicate a very clear identification EpCAM + CTCs, EpCAM- CTCs, and WBCs (The details are shown in Figure 3). The immunofluorescence staining further confirms the advantageous performance of PSINR equipment, and the results also proved that capturing and releasing EpCAM + CTCs and EpCAM- CTCs is completely feasible.

## 4 Disscusion

Liver cancer is considered one of the most heterogeneous tumors, and heterogeneity is the main cause of tumor metastasis, recurrence, and drug resistance (McGranahan and Swanton, 2017; Tellez-Gabriel et al., 2019). However, due to the highly heterogeneous of liver cancer, individualized clinical treatment of liver cancer has great blindness, limitations, and differences, which also poses great challenges to the diagnosis and treatment of liver cancer. The chemotherapy resistance, lack of effective targets, and recurrence and metastasis of liver cancer have always troubled clinical oncologists. Therefore, it is urgent for researchers to explore new ways to decipher the heterogeneity of liver cancer.

CTCs can be regarded as liquid specimens of solid tumors, which have advantages such as easy acquisition, minimal trauma, non-invasiveness, and repeated collection, making them a more ideal source of specimens for clinical testing. In recent years, researchers have developed techniques and methods for specific capture of CTCs based on the principle of combining surface antigens and antibodies of CTCs. However, there were also some stromal CTCs in liver cancer patients, leading to low expression or even lack of EpCAM in some CTCs (Lampignano et al., 2017a). Given the differential expression of EpCAM in CTCs, this provides us with valuable insights into the heterogeneity of liver cancer.

The EpCAM is widely expressed in epithelial tumors, and the EpCAM has a variety of biological functions such as regulating cell proliferation, differentiation, and migration (Amoury et al., 2016; Kubo et al., 2018). Therefore, the differential expression of EpCAM can have an important impact on the biological characteristics of tumor cells, especially the effects of tumor metastasis and recurrence. We utilize a PSINR to specific capture and release the EpCAM + CTCs and EpCAM- CTCs of liver cancer at the same time.

As we all known, the number of EpCAM + CTCs and EpCAM- CTCs is too small, and the capture and release operations may affect the viability of released CTCs. We should strive to increase the quantity and viability of released CTCs, and make subsequent biological analysis or *in vitro* culture possible. The in-depth analysis of releasing CTC will bring greater significance.

Our study is a novel form of exploring the heterogeneity of liver cancer. The heterogeneity of liver cancer is expected to reveal the key biological mechanisms of tumor recurrence, metastasis, and drug resistance. Most importantly, the heterogeneity of liver cancer helps to guide clinical chemotherapy, targeted therapy, and other precision treatments, thereby making personalized treatment of liver cancer more targeted, safe, and effective, and providing new basis for achieving precise personalized treatment of tumors.

## Data Availability

The original contributions presented in the study are included in the article/supplementary material, further inquiries can be directed to the corresponding author.
